# Supplementary material for: Can the application of machine learning to electronic health records guide antibiotic prescribing decisions for suspected urinary tract infection in the Emergency Department?
Source: PLOS Digit Health. 2023 Jun 13;2(6):e0000261. doi: 10.1371/journal.pdig.0000261 (PMC10263340; doi:10.1371/journal.pdig.0000261)
Supplement: S1 Table — (DOCX) [file pdig.0000261.s002.docx]

**S1 Table. Hyperparameter ranges used for model tuning.**

| **Model** | **Parsnip function** | **Parsnip engine** | **Hyperparameter** | **Range** |
| --- | --- | --- | --- | --- |
| Logistic regression | logistic_reg | glm | - | - |
| Logistic regression with Fractional polynomials | logistic_reg | mfp (custom) | - | - |
| Elastic Net | Logistic_reg | glmnet | Penalty | 10**[-4, 0] |
|  |  |  | Mixture | [0, 1] |
| XGBoost | boost_tree | xgboost | Tree depth | [1, 2, …, 20] |
|  |  |  | Mtry | [1, …, n_features] |
|  |  |  | Sample size | [0.2, 0.8] |
|  |  |  | Learning rate | 10**[-3, -0.7] |
|  |  |  | Loss reduction | 10**[-3, -1] |
| Random Forest | rand_forest | randomForest | Trees | [100, …, 1000] |
|  |  |  | Mtry | [1, …, n_features] |
