# Supplementary material for: Can the application of machine learning to electronic health records guide antibiotic prescribing decisions for suspected urinary tract infection in the Emergency Department?
Source: PLOS Digit Health. 2023 Jun 13;2(6):e0000261. doi: 10.1371/journal.pdig.0000261 (PMC10263340; doi:10.1371/journal.pdig.0000261)
Supplement: S2 Table — AUC, Specificity, and NPV when predicting bacterial growth during 10-times repeated 10-fold cross-validation of the development set. (DOCX) [file pdig.0000261.s003.docx]

**S2 Table. Discriminative performance in internal validation.** AUC, Specificity, and NPV when predicting bacterial growth during 10-times repeated 10-fold cross-validation of the development set.

| **Model** | **AUC (95% CI)** | **Specificity (95% CI)** | **NPV (95% CI)** | **p-value** |
| --- | --- | --- | --- | --- |
| All candidate predictors | | | | |
| XGB | 0.808 (0.805-0.811) | 34.9 (33.9-35.9) | 92.4 (92.2-92.7) | - |
| RF | 0.804 (0.802-0.807) | 35.4 (34.5-36.4) | 92.5 (92.3-92.7) | <0.001 |
| E-NET | 0.788 (0.785-0.791) | 29.7 (28.8-30.5) | 91.2 (91.0-91.5) | <0.001 |
| LR | 0.785 (0.782-0.788) | 28.7 (27.8-29.5) | 90.9 (90.7-91.2) | <0.001 |
| LR-FP | 0.781 (0.778-0.784) | 28.3 (27.4-29.2) | 90.8 (90.5-91.1)) | <0.001 |
| Reduced set of predictors | | | | |
| XGB | 0.795 (0.792-0.798) | 34.3 (33.5-35.2) | 92.4 (92.2-92.6) | <0.001 |
| E-NET | 0.773 (0.770-0.777) | 28.6 (27.9-29.4) | 90.8 (90.6-91.0) | <0.001 |
| LR | 0.773 (0.769-0.776) | 28.8 (28.1-29.5) | 90.9 (90.6-91.0) | <0.001 |
| LR-FP | 0.768 (0.765-0.771) | 27.5 (26.7-28.2) | 90.4 (90.2-90.7) | <0.001 |
| RF | 0.767 (0.764-0.770) | 12.2 (11.7-12.6) | 77.5 (76.7-78.4)) | <0.001 |

Specificity and NPV were calculated at a predefined sensitivity of 95%. p-values were obtained via Bayesian generalised linear mixed models [(Benavoli et al. 2017)](https://paperpile.com/c/40MT9z/hCbH).

AUC, area under the receiver operating characteristic; CI, confidence interval; E-NET, elastic net; LR, logistic regression; LR-FP, logistic regression with fractional polynomials; NPV, negative predictive value; RF, random forest; XGB, extreme gradient boosting trees.
