# Supplementary material for: Can the application of machine learning to electronic health records guide antibiotic prescribing decisions for suspected urinary tract infection in the Emergency Department?
Source: PLOS Digit Health. 2023 Jun 13;2(6):e0000261. doi: 10.1371/journal.pdig.0000261 (PMC10263340; doi:10.1371/journal.pdig.0000261)
Supplement: S3 Table — Estimated AUC of LR and XGB using all predictors during external validation, by imputation method. (DOCX) [file pdig.0000261.s004.docx]

**S3 Table. Discriminative performance by imputation method.** Estimated AUC of LR and XGB using all predictors during external validation, by imputation method.

| **Imputation method** | **Model** | |
| --- | --- | --- |
|  | **LR** | **XGB** |
|  | AUC (95% CI) | AUC (95% CI) |
| Mean | 0.796 (0.776-0.817) | 0.813 (0.792-0.834) |
| kNN | 0.796 (0.776-0.817) | 0.814 (0.794-0.835) |
| MICE (M=5) | 0.769 (0.742-0.796) | ***** |

* Not calculated due to computational limitations in the data safe haven within which the analysis was performed.

AUC, area under the receiver operating characteristic; CI, confidence interval; kNN, k-nearest neighbours; LR, logistic regression; MICE; multivariate imputation by chained equations; XGB, extreme gradient boosting trees.
