# Supplementary material for: Can the application of machine learning to electronic health records guide antibiotic prescribing decisions for suspected urinary tract infection in the Emergency Department?
Source: PLOS Digit Health. 2023 Jun 13;2(6):e0000261. doi: 10.1371/journal.pdig.0000261 (PMC10263340; doi:10.1371/journal.pdig.0000261)
Supplement: S4 Table — Top ten variables with the highest AUC when predicting bacterial growth during internal validation of LR and XGB. (DOCX) [file pdig.0000261.s005.docx]

**S4 Table. Feature importance.** Top ten variables with the highest AUC when predicting bacterial growth during internal validation of LR and XGB.

| **LR** | | |  | **XGB** | | |
| --- | --- | --- | --- | --- | --- | --- |
| **#** | **Variable** | **AUC (95% CI)** |  | **#** | **Variable** | **AUC (95% CI)** |
| 1 | UFC bacteria | 0.631 (0.625-0.636) |  | 1 | UFC bacteria | 0.662 (0.657-0.667) |
| 2 | ED diagnosis | 0.603 (0.598-0.608) |  | 2 | UFC white blood cells | 0.608 (0.602-0.614) |
| 3 | UFC epithelial cells | 0.585 (0.577-0.593) |  | 3 | ED diagnosis | 0.603 (0.598-0.609) |
| 4 | UFC white blood cells | 0.581 (0.575-0.587) |  | 4 | UFC epithelial cells | 0.580 (0.573-0.588) |
| 5 | UFC casts | 0.561 (0.556-0.566) |  | 5 | UFC casts | 0.560 (0.555-0.565) |
| 6 | UFC small round cells | 0.555 (0.549-0.562) |  | 6 | UFC small round cells | 0.559 (0.552-0.565) |
| 7 | UFC red blood cells | 0.549 (0.543-0.556) |  | 7 | Age | 0.548 (0.542-0.553) |
| 8 | Age | 0.548 (0.542-0.553) |  | 8 | Missing UFC | 0.546 (0.539-0.554) |
| 9 | Missing UFC | 0.546 (0.539-0.554) |  | 9 | UFC red blood cells | 0.543 (0.535-0.550) |
| 10 | Previous bacteriuria | 0.539 (0.534-0.545) |  | 10 | Previous bacteriuria | 0.539 (0.534-0.545) |

AUC, area under the receiver operating characteristic; CI, confidence interval; ED, emergency department; LR, logistic regression; UFC, urine flow cytometry; XGB, extreme gradient boosting trees.
