# Supplementary material for: Can the application of machine learning to electronic health records guide antibiotic prescribing decisions for suspected urinary tract infection in the Emergency Department?
Source: PLOS Digit Health. 2023 Jun 13;2(6):e0000261. doi: 10.1371/journal.pdig.0000261 (PMC10263340; doi:10.1371/journal.pdig.0000261)
Supplement: S5 Table — Comparison of demography, medical history, and clinical characteristics between training set (before or in 2017) and test set (after 2017). (DOCX) [file pdig.0000261.s006.docx]

**S5 Table. Characteristics of train and test data.** Comparison of demography, medical history, and clinical characteristics between training set (before or in 2017) and test set (after 2017).

|  | **Training set** | | **Test set** | | **p-value** |
| --- | --- | --- | --- | --- | --- |
|  | Summary | Missing % | Summary | Missing % |  |
| Number of visits | 10,352 (100.0) |  | 1,538 (100.0) |  |  |
| **Demographics** | | | | | |
| ≥65 years (%) | 6,142 (51.7) | 0.0 | 5,382 (52.0) | 0.0 | 0.063 |
| Female (%) | 7,851 (66.0) | 0.0 | 6,838 (66.1) | 0.0 | 0.906 |
| Ethnicity (%) |  |  |  |  | 0.035 |
| Asian | 1,342 (13.9) |  | 221 (16.1) |  |  |
| Black | 447 (4.6) |  | 68 (4.9) |  |  |
| White | 7,666 (77.1) |  | 1,019 (74.1) |  |  |
| Other | 434 (4.5) |  | 68 (4.9) |  |  |
| **Comorbidities** | | | | | |
| Charlson comorbidity index (%) |  | 0.0 |  | 0.0 | 0.016 |
| 0 | 6,089 (58.8) |  | 956 (62.2) |  |  |
| 1-2 | 2,482 (24.0) |  | 320 (20.8) |  |  |
| ≥3 | 1,781 (17.2) |  | 262 (17.0) |  |  |
| Cancer (%) | 714 (6.9) | 0.0 | 126 (8.2) | 0.0 | 0.072 |
| Underlying renal condition (%) | 2,134 (20.6) | 0.0 | 304 (19.8) | 0.0 | 0.078 |
| Underlying urological condition (%) | 2,902 (28.0) | 0.0 | 336 (21.8) | 0.0 | 0.462 |
| Renal/urological surgery (%) | 1,985 (19.2) | 0.0 | 256 (16.6) | 0.0 | 0.020 |
| **Hospital activity in prior year** | | | | | |
| Any hospitalisation (%) | 4,910 (47.4) | 0.0 | 667 (44.0) | 0.0 | 0.013 |
| Urine sample taken (%) | 5,097 (49.2) | 0.0 | 623 (40.5) | 0.0 | <0.001 |
| Urine sample positive (%) | 2,476 (23.9) | 0.0 | 306 (19.9) | 0.0 | 0.001 |
| Antibiotics in hospital (%) | 2,497 (24.1) | 0.0 | 366 (23.8) | 0.0 | 0.806 |
| **Presentation in the ED** | | | | | |
| Recorded ED diagnosis (%) |  | 4.1 |  | 18.4 | <0.001 |
| UTI | 4,054 (39.2) |  | 639 (41.5) |  |  |
| UTI symptoms | 1,554 (15.0) |  | 112 (7.3) |  |  |
| Other infection | 1,339 (12.9) |  | 228 (14.8) |  |  |
| Other diagnoses | 2,978 (28.8) |  | 276 (17.9) |  |  |
| Urine flow cytometry (median / IQR) |  |  |  |  |  |
| Bacteria x10^3^/μL | 9.0 (3.0, 24.)] | 13.3 | 3.4 (0.5, 11.1) | 24.9 | <0.001 |
| White blood cells x1/μL | 311 (103, 1154) | 13.3 | 410 (157, 1321) | 24.9 | <0.001 |
| Red blood cells x1/μL | 38.0 (14.0, 151.0) | 13.3 | 24.0 (10.0, 76.5) | 24.9 | <0.001 |
| Epithelial cells x1/μL | 23.0 (8.0, 58.5) | 13.3 | 11.0 (3.0, 32.5) | 24.9 | <0.001 |
| Small round cells x1/μL | 2.0 (1.0, 4.0) | 13.2 | 1.0 (0.0, 3.0) | 24.9 | <0.001 |
| Casts x1/μL | 1.0 (0.0, 2.0) | 13.2 | 1.0 (0.0, 2.0) | 24.9 | 0.021 |
| Crystals x1/μL | 5.0 (2.0, 15.0) | 51.1 | - | 100.0 |  |
| Blood tests (median / IQR) |  |  |  |  |  |
| C-reactive protein mg/L | 27.0 (6.0, 94.0) | 56.4 | 34.0 (8.0, 94.0) | 43.5 | 0.050 |
| White blood cells x10^3^/μL | 10.8 (8.1, 14.5) | 46.3 | 10.8 (8.0, 14.5) | 34.3 | 0.907 |
| Platelets x10^3^/μL | 229 (181, 290) | 46.4 | 238 (190, 302) | 34.5 | <0.001 |
| Creatinine μmol/L | 84.0 (66.0, 119) | 48.0 | 79.5 (64.0, 109) | 33.6 | <0.001 |
| Bilirubin μmol/L | 9.0 (6.0, 14.0) | 54.7 | 9.0 (6.0, 14.0) | 36.5 | 0.989 |
| Alkaline phosphatase IU/L | 87.0 (68.0, 117) | 54.3 | 88.0 (69.0, 117) | 38.3 | 0.789 |
| **Outcome** |  |  |  |  |  |
| Bacterial growth observed | 3,850 (35.5) | 0.0 | 678 (44.1) | 0.0 | <0.001 |

ED, emergency department; IQR, interquartile range.
