# Supplementary material for: Can the application of machine learning to electronic health records guide antibiotic prescribing decisions for suspected urinary tract infection in the Emergency Department?
Source: PLOS Digit Health. 2023 Jun 13;2(6):e0000261. doi: 10.1371/journal.pdig.0000261 (PMC10263340; doi:10.1371/journal.pdig.0000261)
Supplement: S6 Table — Applied Yeo-Johnson transformations and final model coefficients for baseline LR models using all predictors or the reduced set of predictors. (DOCX) [file pdig.0000261.s007.docx]

**S6 Table. Parameters of final LR model.** Applied Yeo-Johnson transformations and final model coefficients for baseline LR models using all predictors or the reduced set of predictors.

|  | **Yeo-Johnson transformation** | | | **LR**  **All predictors** | **LR**  **Reduced predictors** |
| --- | --- | --- | --- | --- | --- |
|  | Lambda | Mean | SD | OR (95% CI) | OR (95% CI) |
| Base probability* |  |  |  |  |  |
| **Demographics** | | | | | |
| Age in years |  |  |  |  |  |
| 18 - 25 |  |  |  | 1 | 1 |
| 25 - 34 |  |  |  | 1.15 (0.95-1.39) | 1.26 (1.05-1.52) |
| 35 - 44 |  |  |  | 0.98 (0.79-1.21) | 1.15 (0.93-1.41) |
| 45 - 54 |  |  |  | 0.82 (0.66-1.02) | 1.06 (0.86-1.30) |
| 55 - 64 |  |  |  | 0.95 (0.77-1.19) | 1.21 (0.98-1.49) |
| 65 - 74 |  |  |  | 0.81 (0.66-1.00) | 1.07 (0.89-1.29) |
| 75 - 84 |  |  |  | 0.95 (0.78-1.15) | 1.29 (1.08-1.54) |
| 85 - 94 |  |  |  | 0.90 (0.74-1.10) | 1.26 (1.05-1.50) |
| 95 - 104 |  |  |  | 1.18 (0.81-1.72) | 1.64 (1.14-2.35) |
| Female |  |  |  | 0.64 (0.57-0.73) | 0.57 (0.51-0.63) |
| Ethnicity |  |  |  |  |  |
| White |  |  |  | 1 |  |
| Asian |  |  |  | 1.04 (0.90-1.21) |  |
| Black |  |  |  | 1.08 (0.86-1.37) |  |
| Other |  |  |  | 1.00 (0.78-1.27) |  |
| Unknown |  |  |  | 0.94 (0.75-1.18) |  |
| **Comorbidities** | | | | | |
| Charlson comorbidity index (linear) |  |  |  | 1.01 (0.98-1.05) |  |
| Cancer |  |  |  | 0.88 (0.70-1.11) |  |
| Underlying renal condition |  |  |  | 0.90 (0.78-1.05) |  |
| Underlying urological condition |  |  |  | 1.04 (0.90-1.21) |  |
| Renal/urological surgery |  |  |  | 1.24 (1.07-1.44) |  |
| **Hospital activity** | | | | | |
| Hospital activity in prior 7 days |  |  |  | 0.93 (0.74-1.16) |  |
| Number of hosp. in prior year |  |  |  | 0.98 (0.94-1.02) |  |
| Number of UTI hosp. in prior 2 years |  |  |  | 1.06 (0.96-1.17) |  |
| Number of ED visits in prior year |  |  |  | 1 (0.97-1.03) |  |
| Number of UTI ED visits in prior 2 years |  |  |  | 1.11 (1.01-1.22) |  |
| Urine culture in prior year |  |  |  | 1.44 (1.26-1.64) |  |
| Positive urine culture in prior year |  |  |  | 0.62 (0.54-0.71) |  |
| Antibiotics in prior year |  |  |  | 1.08 (0.92-1.26) |  |
| **Presentation in the ED** | | | | | |
| Recorded ED diagnosis (%) |  |  |  |  |  |
| UTI |  |  |  | 1 |  |
| Pyelonephritis |  |  |  | 0.77 (0.62-0.95) |  |
| Urosepsis |  |  |  | 1.57 (1.26-1.95) |  |
| Urinary symptoms |  |  |  | 1.90 (1.49-2.42) |  |
| Altered mental status |  |  |  | 1.71 (1.35-2.17) |  |
| Abdominal pain |  |  |  | 2.43 (1.87-3.16) |  |
| Other sepsis |  |  |  | 2.40 (1.82-3.17) |  |
| Lower resp. tract infection |  |  |  | 2.23 (1.73-2.87) |  |
| Other infections |  |  |  | 2.04 (1.60-2.62) |  |
| Other genitourinary conditions |  |  |  | 1.72 (1.24-2.40) |  |
| Other diagnoses |  |  |  | 1.71 (1.51-1.95) |  |
| Urine flow cytometry  (median / IQR) |  |  |  |  |  |
| Bacteria x10^3^/μL | 0.0590 | 11.80 | 3.137 | 0.34 (0.32-0.37) | 0.35 (0.33-0.37) |
| White blood cells x1/μL | -0.0357 | 5.25 | 1.286 | 0.63 (0.60-0.67) | 0.61 (0.58-0.64) |
| Red blood cells x1/μL | -0.2183 | 2.55 | 0.644 | 1.19 (1.13-1.26) | 1.20 (1.13-1.26) |
| Epithelial cells x1/μL | -0.0078 | 3.08 | 1.255 | 1.72 (1.60-1.85) | 1.72 (1.60-1.85) |
| Small round cells x1/μL | -0.3953 | 0.80 | 0.540 | 0.87 (0.82-0.92) | 0.87 (0.82-0.93) |
| Casts x1/μL | -0.6553 | 0.49 | 0.418 | 1.22 (1.14-1.31) | 1.25 (1.16-1.33) |
| Crystals x1/μL | -0.2442 | 1.46 | 0.564 | 1.07 (1.02-1.12) | 1.07 (1.02-1.12) |
| Blood tests (median / IQR) |  |  |  |  |  |
| C-reactive protein mg/L | -0.0497 | 3.01 | 0.806 | 0.96 (0.91-1.01) |  |
| White blood cells x10^3^/μL | 0.0149 | 2.52 | 0.311 | 0.92 (0.87-0.97) |  |
| Platelets x10^3^/μL | 0.4399 | 22.7 | 3.122 | 1.10 (1.05-1.17) |  |
| Creatinine μmol/L | -0.7859 | 1.23 | 0.009 | 1.04 (0.98-1.09) |  |
| Bilirubin μmol/L | -0.4495 | 1.42 | 0.138 | 1.07 (1.01-1.13) |  |
| Alkaline phosphatase IU/L | -0.7054 | 1.36 | 0.012 | 1.00 (0.95-1.05) |  |
| Missing values |  |  |  |  |  |
| All flow cytometry missing |  |  |  | 0.98 (0.83-1.16) | 1.02 (0.87-1.21) |
| Some flow cytometry missing |  |  |  | 0.44 (0.38-0.51) | 0.46 (0.39-0.53) |
| Only casts missing |  |  |  | 1.17 (0.98-1.41) | 1.17 (0.98-1.40) |
| WBC (blood) missing |  |  |  | 0.92 (0.77-1.10) |  |
| Creatinine count missing |  |  |  | 0.95 (0.70-1.28) |  |
| Alk. phosph. and bili. missing |  |  |  | 1.03 (0.81-1.30) |  |
| C-reactive protein missing |  |  |  | 1.04 (0.87-1.25) |  |
| **Time of arrival** |  |  |  |  |  |
| Month |  |  |  |  |  |
| January |  |  |  | 1 |  |
| February |  |  |  | 0.93 (0.70-1.25) |  |
| March |  |  |  | 0.65 (0.44-0.96) |  |
| April |  |  |  | 0.55 (0.32-0.94) |  |
| May |  |  |  | 0.48 (0.24-0.94) |  |
| June |  |  |  | 0.31 (0.14-0.72) |  |
| July |  |  |  | 0.36 (0.14-0.98) |  |
| August |  |  |  | 0.26 (0.08-0.83) |  |
| September |  |  |  | 0.20 (0.05-0.74) |  |
| October |  |  |  | 0.16 (0.04-0.70) |  |
| November |  |  |  | 0.13 (0.02-0.64) |  |
| December |  |  |  | 0.12 (0.02-0.73) |  |
| Day of the year (linear) |  |  |  | 1.01 (1.00-1.01) |  |
| Day of the week |  |  |  |  |  |
| Monday |  |  |  | 1 |  |
| Tuesday |  |  |  | 1.10 (0.93-1.30) |  |
| Wednesday |  |  |  | 1.08 (0.91-1.28) |  |
| Thursday |  |  |  | 1.02 (0.86-1.21) |  |
| Friday |  |  |  | 1.17 (0.98-1.38) |  |
| Saturday |  |  |  | 1.24 (1.04-1.49) |  |
| Sunday |  |  |  | 1.18 (1.00-1.40) |  |
| Time of day (linear) |  |  |  | 1.00 (0.99-1.01) |  |

* For a reference patient with mean levels at each continuous variable.
