# Supplementary figures and images for: Can the application of machine learning to electronic health records guide antibiotic prescribing decisions for suspected urinary tract infection in the Emergency Department?
Source: PLOS Digit Health. 2023 Jun 13;2(6):e0000261. doi: 10.1371/journal.pdig.0000261 (PMC10263340; doi:10.1371/journal.pdig.0000261)

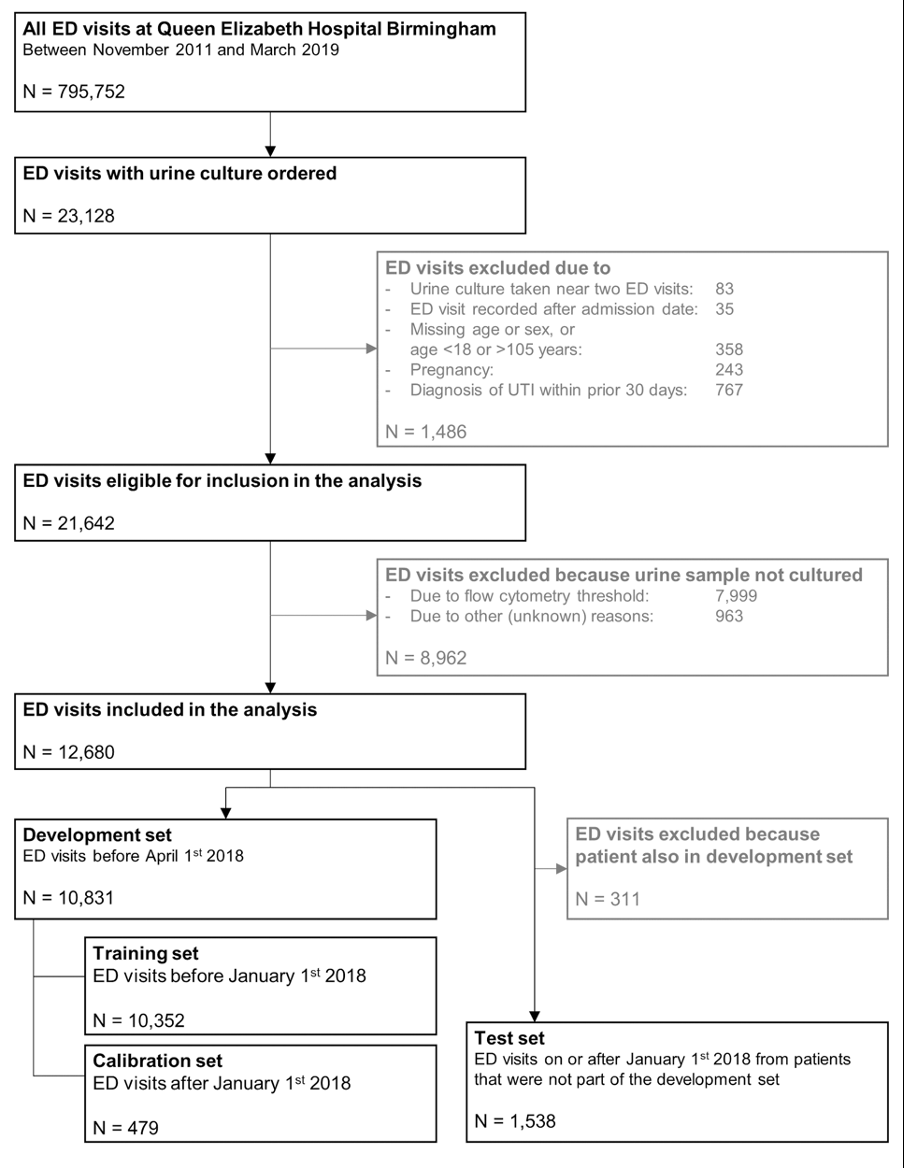

Supplement: S1 Fig — (TIF) [file pdig.0000261.s008.tif]

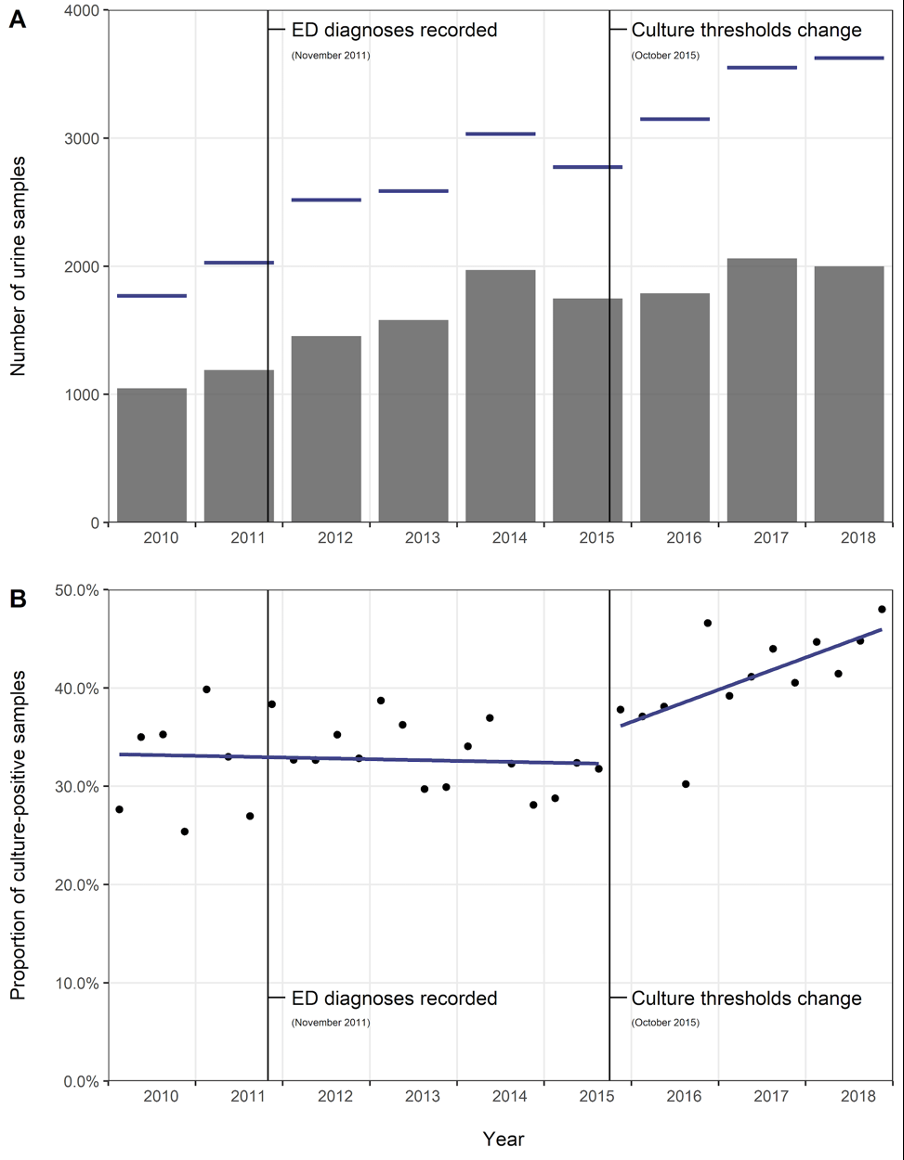

Supplement: S2 Fig — A) Yearly distribution of ED visits with a urine sample sent for microbiological culture (blue lines), and number of ED visits for which the urine sample was ultimately cultured (grey bars). Although visits before November 2011 are presented here to show an overall trend, they were not included in the main analysis since ED diagnoses were not yet recorded for these visits. B) Quarterly proportion of cultured urine samples that showed predominant bacterial growth (black dots) and linear trend (blue lines) before and after the change in urinalysis thresholds in October 2015. (TIF) [file pdig.0000261.s009.tif]

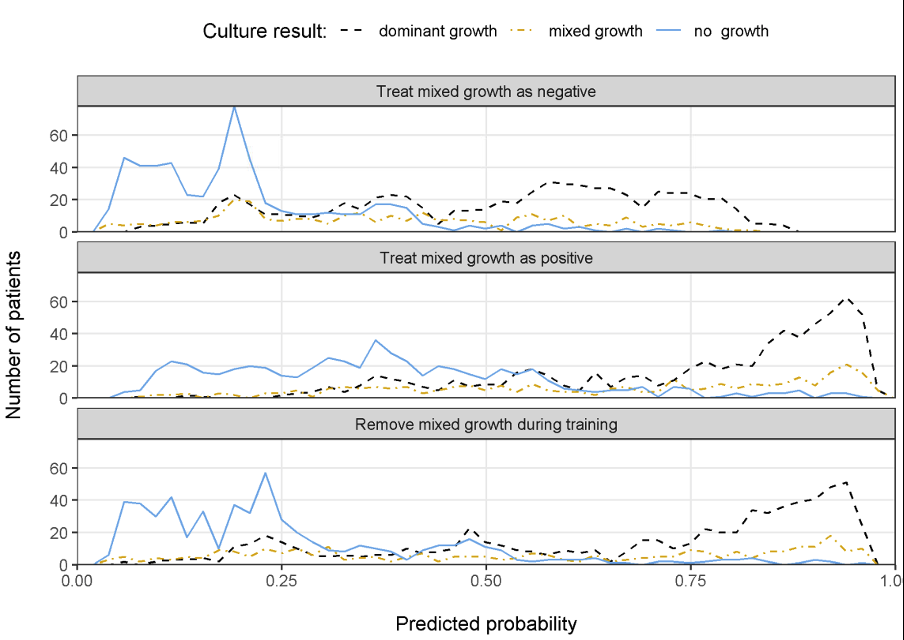

Supplement: S3 Fig — Distribution of model predictions in the test set for samples with dominant growth, mixed growth, and no growth, depending on how mixed growth was treated during model training. (TIF) [file pdig.0000261.s010.tif]
