# Supplementary material for: Can the application of machine learning to electronic health records guide antibiotic prescribing decisions for suspected urinary tract infection in the Emergency Department?
Source: PLOS Digit Health. 2023 Jun 13;2(6):e0000261. doi: 10.1371/journal.pdig.0000261 (PMC10263340; doi:10.1371/journal.pdig.0000261)
Supplement: S1 Text — (DOCX) [file pdig.0000261.s011.docx]

# Systemic antibiotics recommended for UTI

The 2018 prescribing guidelines at Queen Elizabeth Hospital Birmingham recommended the following systemic antibiotics for the treatment of UTI in non-pregnant patients:

- **Lower UTI**
  - First line: nitrofurantoin (oral)
  - Second line: trimethoprim (oral)
- **Pyelonephritis**
  - First line: amoxicillin (iv), co-amoxiclav (oral), gentamicin (iv)
  - Second line: ciprofloxacin (oral), vancomycin (if history of UTI or suspected MRSA; iv), ertapenem (if history of UTI or suspected ESBL; iv)
- **(Uro)sepsis**
  - First line: co-amoxiclav (iv), gentamicin (iv)
  - Second line: ceftriaxone (iv), ciprofloxacin (iv), vancomycin (iv), piperacillin- tazobactam (iv), meropenem (if suspected ESBL or septic shock; iv)

Note that some of the above antibiotics are indicated in combination (e.g. co-amoxiclav and gentamicin as first line treatment for urosepsis). This was not enforced in this study and any prescription of one or more of the above antibiotics was considered as potential treatment for UTI for the purposes of this study.
