# Supplementary material for: Can the application of machine learning to electronic health records guide antibiotic prescribing decisions for suspected urinary tract infection in the Emergency Department?
Source: PLOS Digit Health. 2023 Jun 13;2(6):e0000261. doi: 10.1371/journal.pdig.0000261 (PMC10263340; doi:10.1371/journal.pdig.0000261)
Supplement: S2 Text — (DOCX) [file pdig.0000261.s012.docx]

# Missing data

EHR data is primarily recorded with patient management in mind. Information is only recorded if measuring *and* recording the information in the EHR system was prescribed by clinical guidelines or deemed necessary by the healthcare personnel in charge of patient care. Missing data is therefore ubiquitous when using EHR data for research. Negative information — i.e., information on the absence of an event — is seldom recorded explicitly, making it difficult to distinguish failure to record a disease from a genuine absence of disease. For example, a record of renal disease in a patient's medical history explicitly implies that a diagnosis has previously been made. Absence of renal disease, on the other hand, is usually implicitly signalled by an *absence* of diagnosis codes. As a consequence, we were unable to distinguish between cases in which a patient truly did not have a disease, and cases where the patient did suffer from the disease but the disease was not recorded, either because it hasn't been diagnosed yet or because the diagnosis was not captured in EHR records. For variables that describe the presence or absence of an event — i.e., those related to comorbidity diagnoses and past healthcare activity — we therefore assumed that an absence of a record meant that the event did not take place.

Missingness could be ascertained, however, in clinical information relating to general patient characteristics and clinical observations — i.e., demographic information, urine flow cytometry results, vital signs, and blood tests. For example, a missing record of heart rate can be unambiguously interpreted. We imputed likely values of these variables using several common imputation methods. These methods differed in their computational complexity but also statistical capability of faithfully reflecting the uncertainty caused by missing data. They may therefore differ in their impact on model performance. The following three imputation methods were applied to assess their impact on model performance:

**Mean imputation and missing indicators:** For mean imputation, each missing numerical value was replaced by the mean observed value in the training set. For categorical variables, missing values were assigned to a "missing" category. To allow the models to distinguish between patients with observed values and patients for whom the value was imputed with the mean, the models were additionally supplied with dummy variables that indicated the presence (0) or absence (1) of the value in the original, unimputed data.

**k-nearest neighbours imputation**: For k-nearest neighbours (KNN) imputation, each patient was matched with k patients most similar to him or her. Similarity between observations was estimated using Gower's distance, which is able to handle both continuous and categorical predictors [(Gower 1971)](https://paperpile.com/c/40MT9z/wTyb). Once the k neighbours were identified for a patient, his or her missing numerical values were imputed with the mean value among the k neighbours and categorical values were imputed with the mode. A value of k=5 was chosen for this analysis.

**Multiple imputation using multivariate imputation by chained equations**: All previous imputation methods produce only a single imputation for each missing value. The prediction model would treat those values as if they were actually observed, ignoring the inherent uncertainty involved in the imputation process [(White et al. 2011)](https://paperpile.com/c/40MT9z/GsnJ). Multiple imputation addresses this issue and imputes each missing value with M random draws from an imputation model — e.g., linear regression or predictive mean matching — resulting in M imputed datasets. A separate prediction model was then fit to each imputed dataset, and the M predictions averaged across datasets. By considering multiple plausible imputations, multiple imputation actively accounts for the uncertainty surrounding the imputation process. Imputation was performed using the multivariate imputation by chained equations (MICE) algorithm with predictive mean matching (continuous variables), logistic regression (binary variables), and multinomial regression (categorical variables) [(White et al. 2011; van Buuren 2018)](https://paperpile.com/c/40MT9z/GsnJ+uVwo). Five datasets were imputed, and the algorithm was run for 10 iterations to achieve approximate convergence. All variables were assumed to be missing at random, meaning that the probability of a value being missing depended only on the values of observed covariates. An example of this missingness mechanism would be if creatinine were less frequently measured in younger, otherwise healthy patients, and the probability of it being measured only depended on age and comorbidities (which are covariates in the data). Due to computational restrictions in the safe haven in which the analysis was performed, multiple imputation was only performed for logistic regression models.

Imputation was performed separately in each resampled dataset (i.e., in each iteration of each [repeated] 10-fold cross validation). Each imputation strategy was trained on the split’s training data (i.e., the combined 9 training folds of the iteration) and applied without retraining to the split’s test data (i.e., the held-out test fold of the iteration). For multiple imputation, we included the outcome as a predictor in the imputation model as recommended [(Moons et al. 2006)](https://paperpile.com/c/40MT9z/ixvk). Since the outcome won't be available during model deployment — i.e., in real-time on the hospital ward — a second set of multiple imputed datasets was imputed *without* the outcome which was then used on the held-out test fold. This ensured that the evaluation faithfully reflected the eventual intended use of the model [(Wood et al. 2015; Rockenschaub et al. 2020)](https://paperpile.com/c/40MT9z/J0HE+TQMV).
