# Supplementary material for: Can the application of machine learning to electronic health records guide antibiotic prescribing decisions for suspected urinary tract infection in the Emergency Department?
Source: PLOS Digit Health. 2023 Jun 13;2(6):e0000261. doi: 10.1371/journal.pdig.0000261 (PMC10263340; doi:10.1371/journal.pdig.0000261)
Supplement: S3 Text — (DOCX) [file pdig.0000261.s013.docx]

# Testing differences in model performance

Model performance may depend on the exact case mix in the training and test sets. We therefore evaluated model performance using resampling techniques in both internal validation (repeated cross-validation) and external validation (bootstrap).

Cross-validation was chosen over bootstrap during internal validation, since the (optimism-adjusted) bootstrap originally proposed in [(Rockenschaub et al. 2020)](https://paperpile.com/c/40MT9z/TQMV) was found inadequate to prevent overfitting for very flexible models such as random forests and gradient boosting trees. To account for the dependence introduced within a single cross-validation split (i.e., a sample that is in fold 1 cannot be in fold 2), differences in model performances were tested for statistical significance using Bayesian generalised linear mixed models estimated via Markov Chain Monte Carlo sampling with four chains of 2,000 warm-up iterations and 2,000 sampling iterations [(Benavoli et al. 2017)](https://paperpile.com/c/40MT9z/hCbH).

Since external validation did not require additional model fitting on the test data (the model is already fit on the entire training data), we chose simple bootstrapping to obtain an estimate of the variability of our performance estimates in the test data. These bootstraps do not suffer from the same dependence that might arise from repeated cross-validation. Confidence intervals were therefore estimated directly from 1,000 bootstraps as the proportion of bootstraps in which model A achieved higher performance than model B, multiplied by two to account for the two-sided nature of our hypothesis.
