# Supplementary material for: Can the application of machine learning to electronic health records guide antibiotic prescribing decisions for suspected urinary tract infection in the Emergency Department?
Source: PLOS Digit Health. 2023 Jun 13;2(6):e0000261. doi: 10.1371/journal.pdig.0000261 (PMC10263340; doi:10.1371/journal.pdig.0000261)
Supplement: Attachment — Submitted filename: Review - Can the application of machine learning to electronic health records guide antibiotic prescribing. PDIG-D-22-00301.docx [file pdig.0000261.s014.docx]

**Review – PDIG-D-22-00301**

**Can the application of machine learning to electronic health records guide antibiotic prescribing decisions for suspected urinary tract infection in the Emergency Department?**

The article discusses the possibility of improving the prescription of antibiotics to patients with a high risk of urinary tract infection using prediction models based on data mining algorithms.

Comments, article weaknesses and suggestions for big improvements:

1) The data analysis methodology described in the article is thorough and comprehensive, the researchers made extensive use of sensitivity analyses. The reason for such analysis is the robustness of the proposed models, the authors should mention it in the text.
2) I would suggest discussing false positive/false negative results together with sensitivity/specificity results and connecting them to the main objective of the proposed research.
3) The presentation of the results and the description of experiments in text in many situations are confusing and not uniform and require repeatedly returning and re-reading. All these parts must be carefully reworked and rewritten again.
4) In the discussion chapter, the authors should explain the meaning of different accuracy metrics in the context of the medical problem and not repeat AUC, sensitivity, etc. as in the results chapter.
5) The implementation part should be more detailed and give, as far as I can understand practical recommendations.
6) Provide ranges of accuracy metrics that are "good enough" to call it "improvement".
7) Page 18 – there is no "p-value test for difference" in statistics. Rephrase.
8) You cannot use non-significant results (p-value>0.05) as a proven finding.
