# Supplementary material for: Can the application of machine learning to electronic health records guide antibiotic prescribing decisions for suspected urinary tract infection in the Emergency Department?
Source: PLOS Digit Health. 2023 Jun 13;2(6):e0000261. doi: 10.1371/journal.pdig.0000261 (PMC10263340; doi:10.1371/journal.pdig.0000261)
Supplement: Attachment — Submitted filename: UTI_ED_author_reply.docx [file pdig.0000261.s015.docx]

April 5^th^, 2023

**RE: PDIG-D-22-00301: manuscript "Can the application of machine learning to electronic health records guide antibiotic prescribing decisions for suspected urinary tract infection in the Emergency Department?"**

Dear Dr Wang,

Thank you for the opportunity to reply to the constructive comments made by the reviewers. On behalf of my co-authors, I have submitted a revised manuscript via the online portal. All authors named on the paper have reviewed and approved this revised manuscript.

In the pages following this letter, we respond to each of the points raised by the reviewers. We amended the manuscript to take account of the reviewers’ feedback and submitted a version of the manuscript with and without tracked changes. The manuscript is also formatted according to PLOS Digital Health’s style requirements.

We believe that the suggested changes have improved the impact and readability of the manuscript and hope you will now find it acceptable for publication in PLOS Digital Health.

Yours sincerely,

Patrick Rockenschaub

Institute of Health Informatics

University College London

222 Euston Rd, NW1 2AB, London, UK

Reply to Reviewer #1

**Comment #1.1** +ve points:

-this paper studies an important problem.

-Overall, the paper is well written, clear and easy to follow.

- Sufficiently detailed and provides thorough empirical analysis of the results, including calibration and sensitivity analysis of the results

- Code is released publicly

**Reply #1.1** We thank the reviewer for the positive feedback and for noting the importance of publishing the analysis code alongside the manuscript.

**Comment #1.2** Use of proportions of bootstaps for the p-value is bit new to me. Could you provide some references/explanations.

**Reply #1.2** We assume that the reviewer is familiar with the concept of bootstrap but briefly reiterate it here for sake of completeness. During bootstrap, we repeatedly resample by drawing from the original data with replacement (in our case 1,000 times). At the end, we have many, slightly different datasets of the same size N. For each of these datasets, we can calculate the quantity of interest, say AUC. DiCiccio and Efron (1996) show how to derive valid confidence intervals from the percentiles of these 1,000 AUCs. This exact approach is for example implemented in the ci.auc function of the pROC R package.

Efron and Tibshirani (1993) further describe how the same percentiles can be used for hypothesis testing. We employed exactly this to test for a difference in model performance. The quantity of interest in our case isn’t the AUC directly, but rather the difference between the AUC of model M and our best performing model, which was an XGBoost. By evaluating both M and XGBoost on each bootstrapped test set, we get a set of paired AUCs per bootstrapped test set. The difference is AUC_xgboost – AUC_M. The one-sided p-value described the proportion of bootstraps for which this difference was < 0, i.e., AUC_xgboost < AUC_m.

For clarity, we added the following references to the manuscript:

DiCiccio TJ and Efron B. (1996). Bootstrap Confidence Intervals. Statistical Science, 11(3), 189–212.

Efron B and Tibshirani R. (1994). An Introduction to the Bootstrap. Boca Raton, FL: Chapman & Hall/CRC.

**Comment #1.3** Authors mentioned different types of tests for checking the statistical significance. If you could elaborate (maybe in the supplementary material) when and where were those used then that will be helpful for the readers.

**Reply #1.3** We assume the reviewer refers to our use of t-tests, Wilcoxon rank-sum tests, and Chi-squared tests. These tests were exclusively used for the bivariate tests presented in Table 1. We changed the following sentence in the methods to make this clearer:

“Differences in variable distribution by culture status were tested via t-test (normal continuous variables), Wilcoxon rank-sum test (non-normal continuous variables), and χ2 test (categorical variables).“

We also added the following footnote to Table 1:

“p-values refer to the result of a t-test for difference in means (normal continuous variables), a Wilcoxon rank-sum test for difference in means (non-normal continuous variables), or a χ2 test for independence (categorical variables).”

**Comment #1.4** Authors mention that they used 30 random runs for hyperparameter tuning. Please also provide the name and range of values (maybe in the supplementary materials) used.

**Reply #1.4** Thank you for pointing out the missing hyperparameter ranges. We added a corresponding table to the supplement (Supplementary Table 1).

**Comment #1.5** What decision thresholds were used and how were they calculated?

**Reply #1.5** Thresholds were chosen to achieve a sensitivity of 95% for each model. This value was prespecified in the protocol and was considered the minimum sensitivity necessary for using such a model in clinical practice. We changed the description in the text to:

“Thresholds for the calculation of specificity and NPV were chosen such that a predefined sensitivity of 95% was achieved.”

Reply to Reviewer #2

**Comment #2.1** The data analysis methodology described in the article is thorough and comprehensive, the researchers made extensive use of sensitivity analyses. The reason for such analysis is the robustness of the proposed models, the authors should mention it in the text.

**Reply #2.1** We thank the reviewer for the positive feedback and for acknowledging the importance of sensitivity analyses. Following the suggestion, we highlighted the role of sensitivity analysis for achieving robust findings by changing the following sentence in the statistical analysis section:

“In addition to the main analysis, we performed a broad set of sensitivity analyses to assess the robustness of our best model in specific situations and patient subgroups and to determine if there may be scope to deploy the model in clinical practice.”

**Comment #2.2** I would suggest discussing false positive/false negative results together with sensitivity/specificity results and connecting them to the main objective of the proposed research.

**Reply #2.2** We agree that this would be a nice way to discuss the results and tie them to the study objectives. We have therefore added/adapted the following sentences to the discussion (partially overlapping with reply #2.4):

“For example, if the model were used, it would have correctly identified 95% of samples which later showed bacterial growth while ruling out bacterial growth early in 36.6% of ultimately culture-negative samples. Of those samples that were flagged as likely negative, 90.3% were correctly classified and did not exhibit bacterial growth during culture. This highlights the potential of data-driven models to aid the diagnosis of UTI and to reduce laboratory cost if clinical parameters are used in addition to cytometry to select which urines are cultured.”

**Comment #2.3** The presentation of the results and the description of experiments in text in many situations are confusing and not uniform and require repeatedly returning and re-reading. All these parts must be carefully reworked and rewritten again.

**Reply #2.3** We are sorry to hear that the reviewer found our explanations difficult to follow. The manuscript contains a wide range of analyses supporting our claims, which may make it more challenging to read. Yet, we feel it necessary to give sufficient detail to allow replication of all our findings. To make the manuscript easily accessible to clinicians, we ensured that the methods section follows a format common to medical journals: data and study population, outcome, predictors, and statistical analysis. The statistical analysis section is further logically divided into a summary of the patient characteristics, model development and evaluation, and sensitivity analyses. The same structure is mirrored in the results section.

To further highlight this logical connection between methods and results, we now added subheadings to the statistical analysis section and aligned them with subheadings in the results section. We also changed to a more verbose writing style in the results section to ease interpretation. We hope that these changes will make the manuscript more readable.

**Comment #2.4** In the discussion chapter, the authors should explain the meaning of different accuracy metrics in the context of the medical problem and not repeat AUC, sensitivity, etc. as in the results chapter.

**Reply #2.4** We agree with the reviewer that the discussion should focus on the clinical implications. We changed the discussion accordingly and avoided the repetition of accuracy metrics in the discussion section, focusing on their clinical meaning instead (see also reply #2.2).

However, we believe that the use of accuracy metrics was warranted in two cases: 1) the reporting of our peak AUC of 0.815 in the first paragraph, which summarises one of the main findings of our paper, and 2) the comparison to model performances achieved in US (AUC 0.904) and Swiss (AUC 0.930) data. In these two cases, we maintained the use of numeric accuracy metrics.

**Comment #2.5** The implementation part should be more detailed and give, as far as I can understand practical recommendations.

**Reply #2.5** We added a new paragraph to the section on clinical, policy and research implications, discussing how a model like ours might be best implemented in clinical practice:

“We suggest that our model may be embedded within the laboratory workflow. Assuming all relevant clinical data have been recorded at the time of urine sample submission and are readily available within the electronic patient record, the laboratory can use this data and our model to rate the results of flow cytometry and provide rapid feedback to ED clinicians. The results should be reported back to clinicians in such a way that it helps them decide on the likelihood of UTI and choice of antibiotic treatment. On-going education of clinical personnel and audit of the process will be necessary for a successful implementation.”

We made several further changes to the manuscript to increase detail regarding the implementation of the model. We:

- clarified in the abstract and discussion how the model is to be used “to improve UTI diagnosis and thus guide antibiotic prescribing decisions”.
- described in the discussion how the model may be used in the laboratory to “select which urines are cultured”.
- highlighted “the importance of understanding variation in laboratory processes, which can have a major impact on the implementation of ML models in clinical practice.”

**Comment #2.6** Provide ranges of accuracy metrics that are "good enough" to call it "improvement".

**Reply #2.6** While we see the appeal of categorising accuracy into “good enough” and “not good enough”, we do not believe that this would aid the interpretation of our results. From a statistical point of view, any accuracy that exceeds our proxies of clinical performance by a statistically significant margin may be called an “improvement”. Looking at Table 4, this would approximately be the case for any accuracy >60%. From that point of view, our presented results would be “good enough”.

However, whether the model would truly improve clinical practice will depend on a myriad of things, including baseline clinical performance, how good our proxies of clinical performance reflect decision making, and the consequences of false positives (e.g., unnecessary antibiotic prescribing) and false negatives (e.g., lack of safety netting). These factors will inadvertently differ by country or even by hospital (see also reply #2.5). Suggesting an overall accuracy threshold or difference would inevitably discount these important considerations.

Instead of providing an arbitrary threshold for improvement, we therefore opted to provide a detailed description of the variability around our results, overall and within subgroups. This allows us to highlight the factors that influenced model performance in our data. Ideally, we thus provide readers with a sense of how a model like ours would fare at their institution and whether it may be considered “good enough” for their purposes.

**Comment #2.7** Page 18 – there is no "p-value test for difference" in statistics. Rephrase.

**Reply #2.7** We thank the reviewer for pointing out this poor choice of wording. “test” here was used as a verb, which may lead to confusion. We changed this to:

“p-values indicate a difference in AUC compared to the top performing model…”

**Comment #2.8** You cannot use non-significant results (p-value>0.05) as a proven finding.

**Reply #2.7** We are not entirely certain which p-values are meant but we assume that the reviewer refers to the p-values quantifying any differences by ED diagnosis (p=0.210) and by ethnicity (p=0.153), as these are the only p-values > 0.05 that are mentioned in the text. Our intent here was to highlight the possible direction of difference in groups while still prominently reporting the clear lack of statistical significance of these differences. We acknowledge that this may not have been the best way of representing these findings and changed the sentences to:

“There was no significant difference in performance for patients with a record of lower UTI, pyelonephritis, urosepsis, or UTI symptoms (AUC 0.797, 95% CI 0.765-0.828; p=0.210; Table 3), and no evidence that performance varied by ethnicity (AUC 0.831, 95% CI 0.780-0.873; p=0.153).”
